# Sonological predictors of complications of percutaneous renal biopsy—a prospective observational study

**DOI:** 10.1007/s11845-024-03753-y

**Published:** 2024-07-12

**Authors:** Shruti Bhattacharya, Shankar Prasad Nagaraju, Ravindra Attur Prabhu, Dharshan Rangaswamy, Indu Ramachandra Rao, Mohan V. Bhojaraja, Srinivas Vinayak Shenoy

**Affiliations:** Department of Nephrology, Kasturba Medical College, Manipal, Manipal Academy of Higher Education, Manipal, Karnataka 576104 India

**Keywords:** Haematoma surveillance, Renal biopsy complications, Renal sonological parameters

## Abstract

**Abstract:**

Percutaneous renal biopsy, although essential for renal disease diagnosis, is associated with a number of post-biopsy complications ranging from gross haematuria to AV fistula to death. In this study, we carried out an active haematoma surveillance and attempted to correlate renal sonological parameters—kidney length, renal parenchymal changes, renal cortical and parenchymal thickness for their potential use in prediction of post-renal biopsy complications.

**Methods:**

This was a prospective study done from April 2022 to April 2023 on all adult patients undergoing native or transplant kidney biopsy. Baseline clinical, laboratory and renal sonological parameters were noted prior to biopsy. USG-guided renal biopsy was done and any haematoma at 0 h, 12 h and 24 h post-biopsy noted. Biopsy complications including need for any interventions were noted.

**Results:**

Out of the 240 patients enrolled in the study, 58.3% experienced post-biopsy complications. Among these, 5% of patients encountered major complications, with 3.33% necessitating medical intervention following renal biopsy procedures. A high percentage, 98.89%, exhibited hematoma formation within 12 h post-biopsy. Furthermore, our analysis revealed that a hematoma size exceeding 1.2 cm at the 12-h mark exhibited a sensitivity of 100% and specificity of 71% in predicting the need for blood transfusion. Renal parenchymal changes were the most reliable sonological parameters for predicting post-biopsy complication on multivariate analysis.

**Conclusion:**

The incidence of major complications requiring interventions following renal biopsy is notably low. Our study highlights the significance of renal sonological characteristics, including parenchymal thickness, cortical thickness and parenchymal changes, in predicting these complications. Furthermore, we emphasize the utility of hematoma surveillance immediately post-biopsy and at the 12 h, as a valuable tool for predicting the necessity of post-biopsy interventions. This approach can aid in efficiently triaging patients and determining the need for further observation post-renal biopsy.

## Introduction

Percutaneous renal biopsy remains unparalleled in its ability to diagnose kidney diseases despite great advances in non-invasive tests. The information it offers not only helps in diagnosis and modification of treatment but also in prognostication of the disease for both native and transplant kidney [1].

Although an essential diagnostic procedure, renal biopsy should only be performed when appropriately indicated, as this procedure is associated with a number of complications. The incidence of post-renal biopsy complications ranges from 13 to 34%, including bleeding complications, arterio-venous fistulas, abscesses, urinary tract obstruction and even death [2]. More complications are observed among people with coagulation abnormalities, small kidneys and renal dysfunction. Several risk factors have been observed to predict post-renal biopsy bleeding—like older age, hypertension, lower platelet counts, more passes of the biopsy needle, lower glomerular filtration rate (GFR) and abnormal coagulation tests [3–7].

Sonologically determined kidney parameters like kidney length, renal parenchymal changes, renal cortical and parenchymal thickness are postulated to show a relationship with renal function in chronic kidney disease (CKD) patients. However, very few studies have looked into these aspects [8, 9]. In this study, we have attempted to correlate sonological parameters, along with the other traditional risk factors and haematoma surveillance, for their potential use in prediction of post-renal biopsy complications. This may better help us monitor at-risk patients for early identification of post-biopsy complication in them.

## Methodology

This was a prospective observational study of consecutive renal biopsies of adults > 18yrs age between April 2022 to April 2023, at the Department of Nephrology, Kasturba Medical College and Hospital, Manipal. Data of patients who were undergoing renal biopsy for second or more times during the study period were excluded from the analysis and only the first entry was taken.

The primary objective of the study was the correlation of renal sonological parameters like bipolar kidney length, cortical thickness, parenchymal thickness and renal parenchymal changes with the occurrence of post-renal biopsy complications. The secondary objectives were estimation of post-renal biopsy complications, haematoma surveillance with correlation of haematoma size with other biopsy complications and correlation of clinical and laboratory factors associated with occurrence of post-renal biopsy complications.

### Ethical clearance

Institutional Ethics committee clearance was obtained prior to the study (IEC no 454- 2021) and was registered under the Clinical Trial Registry of India (CTRI) with reference no of CTRI/2022/02/039938.

Baseline patient clinical and laboratory parameters including age, sex, comorbidities (diabetes and hypertension), blood pressure at the time of biopsy, serum creatinine, blood urea, complete blood count, coagulation parameters and proteinuria were noted. Blood pressure was controlled to < 160/90 mm Hg prior to the biopsy. Haemoglobin was kept at > 8 g/dl prior to the biopsy, with blood transfusions if required. Biopsy was performed only once the platelet count was > 100,000 cells/cc and coagulation profile was normal. Aspirin and clopidogrel were discontinued for 5 days prior to biopsy and anticoagulants (warfarin, heparin, novel oral anticoagulant (NOAC)) were stopped as indicated. Patients with serum creatinine > 5 mg/dl were optimized by haemodialysis prior to renal biopsy which was as per our department protocol. Intranasal desmopressin at a dosage of 20 mcg was given as per discretion of the consultant depending on availability and when the creatinine was elevated.

### Sonological evaluation

**Fig. 1:**
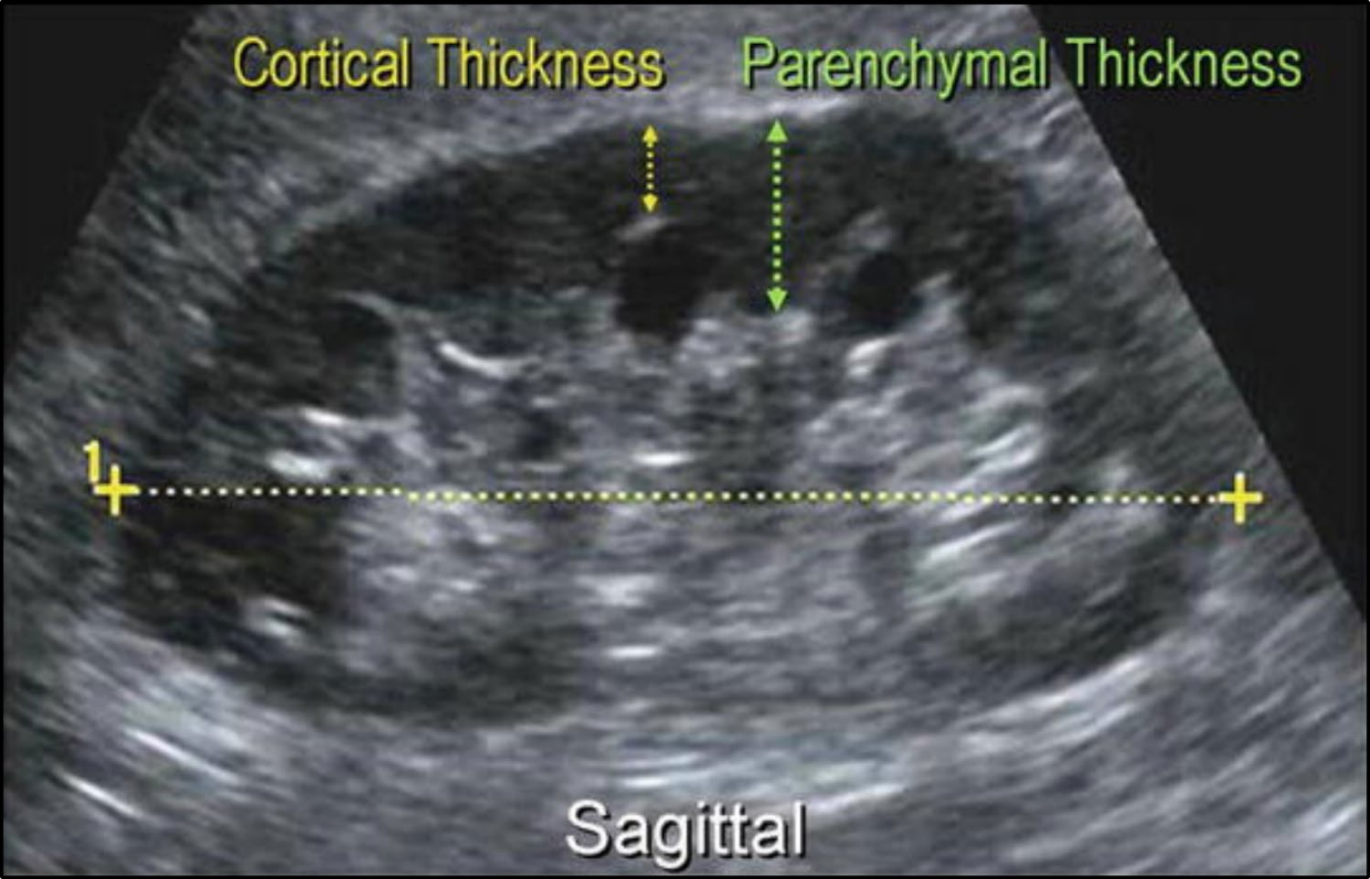
Measurement of renal cortical thickness, renal parenchymal thickness and kidney bipolar length by ultrasonography (Courtesy Campbell-Walsh urology 11th edition)[10]

A screening USG was done prior to the procedure and the bipolar kidney length, renal cortical thickness, renal parenchymal thickness and renal parenchymal changes were noted (Fig. 1).

**Table:** 

Parameter measured	Method of measurement
Renal cortical thickness	Measured by ultrasound in the sagittal plane over a medullary pyramid, perpendicular to the capsule [11]
Renal parenchymal thickness	Measured as the distance between the cortex and perirenal fat interface and the sinus-pyramidal apex interface in the sagittal plane during renal ultrasound [12]
Renal bipolar length	Measured by ultrasound in the sagittal view with the patient in the supine position. The maximum length of each kidney was measured from the upper pole to the lower pole [12]

Renal parenchymal changes: The renal cortex was compared to the liver/spleen and graded as follows [13]:

1. Grade 0—normal-sized kidney, cortical echogenicity less than that of liver/spleen, with well-maintained cortico-medullary differentiation.
2. Grade 1—normal-sized kidney, cortical echogenicity same as that of liver/spleen, with maintained cortico-medullary differentiation.
3. Grade 2—normal-sized kidney, cortical echogenicity more than that of liver/spleen, with decreased cortico-medullary differentiation.
4. Grade 3—reduced renal length, cortical echogenicity more than that of liver/spleen, with poorly maintained cortico-medullary differentiation.

### Biopsy procedure and haematoma surveillance

Kidney biopsy was either performed by a consultant in nephrology or by a fellow in nephrology under the supervision of the consultant, under real-time ultrasonographic guidance with an 18-gauge end-cut spring mounted needle. Usually, two cores were taken for the biopsy, and the number of needle passes taken was recorded. If any haematoma was formed immediately post-biopsy, the size of the haematoma measured along the sagittal plane along its longest diameter was noted. All patients were monitored for 24 h after kidney biopsy. Patients were monitored closely for gross haematuria and flank pain. Haemoglobin and haematocrit levels were monitored at 6 h and 24 h post-biopsy. Blood pressure and heart rate were monitored hourly for 4 h post-biopsy. A follow-up ultrasound was done for all patients at 12 h and 24 h post-biopsy to look for haematoma. Complications such as formation of subcapsular perinephric haematoma, need for blood transfusion, hypotension requiring resuscitation with intravenous fluids or inotropes and any invasive procedure (angiography, nephrectomy) done post-biopsy during the course of the study were recorded.

The renal biopsy report components suggestive of chronicity such as number of obsolescent glomeruli and interstitial fibrosis and tubular atrophy (IFTA) were correlated with the biopsy complications. Adequacy of the biopsy sample in our study was defined as a minimum of 8 glomeruli for native kidney biopsy as per Fogo et al. [14] and a minimum of 10 glomeruli for allograft biopsy as per Geldenhuys et al. [15]

### Outcome measures

The primary outcome was post-biopsy complications which were classified as minor or major. Minor complications included gross haematuria or perinephric haematoma < 5 cm in size on ultrasound imaging or haematocrit drop > 10% not requiring an intervention and Flank pain. Major complications included any intervention—surgical, radiological or blood transfusion, perinephric haematoma > 5 cm in size, AV aneurysm, hypotension that required higher level of nursing care or need for intravenous fluid or vasopressor support.

### Statistical methods

Data was recorded in Microsoft Excel. Continuous variables were expressed as means ± standard deviation (SD), median {interquartile range (IQR)} and range according to normality. Categorical data were summarized in terms of frequencies and percentages. The chi-square test was used to compare categorical data, while the *t*-test or Mann–Whitney *U* test was used to compare continuous data based on normality. Renal sonological parameters that were significant in univariate analysis were put through a binary logistic regression model for their association with complications. A receiver operating characteristic (ROC) curve was plotted for prediction of pos-biopsy haematoma size done as part of surveillance to occurrence of complications. Statistical analysis was carried out using SPSS version 20 (IBM Corp., Armonk, NY, USA). A *p*-value < 0.05 was taken as statistically significant.

## Results

**Fig. 2:**
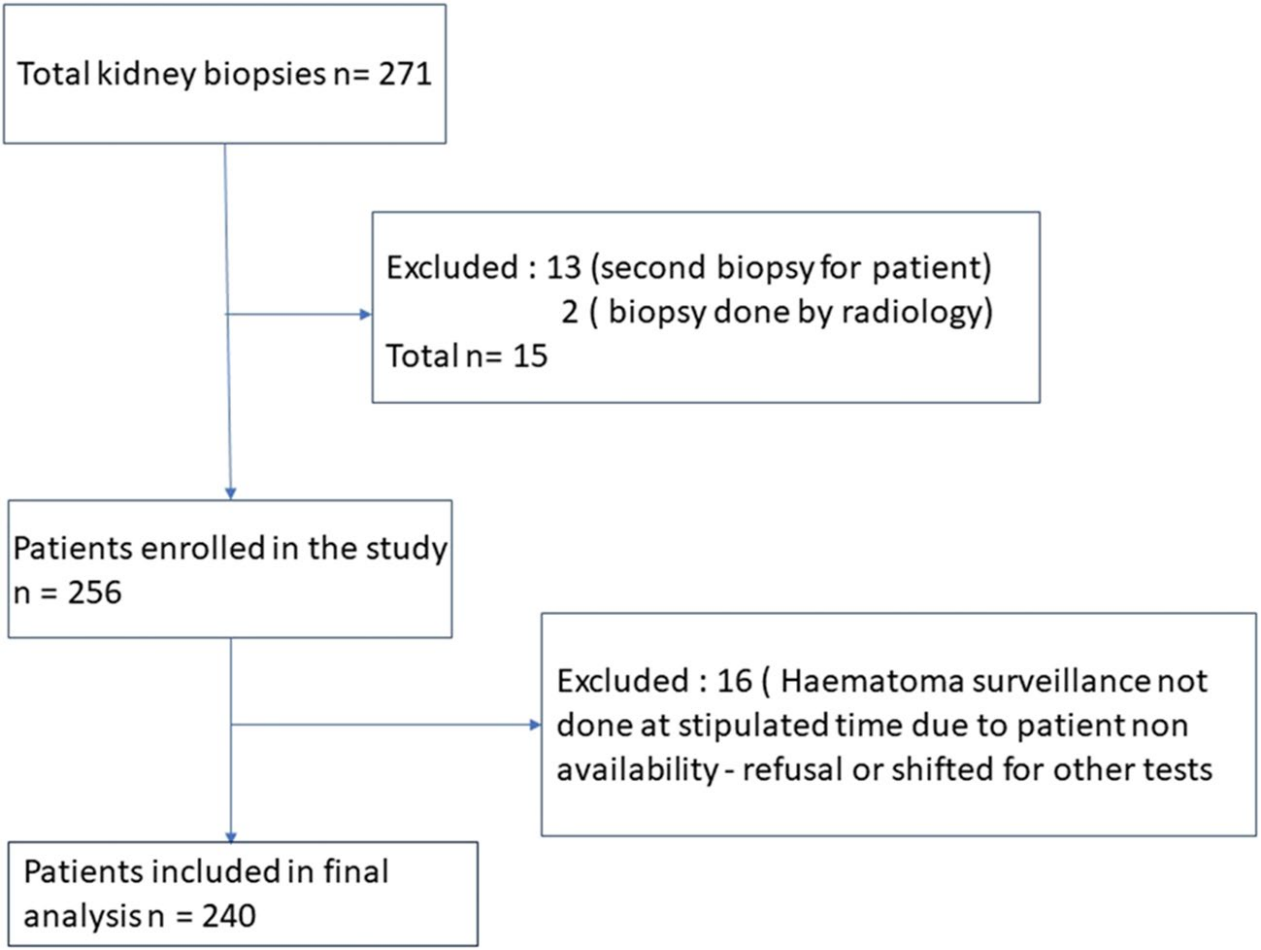
STROBE—patient enrollment flowchart

A total of 240 patients completed the study (Fig. 2). The mean age of patients was 44.62 ± 14.72 years, with 62.9% being male. 86.7% were native kidney biopsies. Seventy-two percent of the biopsies had adequate no. of glomeruli. The most common histopathological diagnosis was IgA nephropathy followed by diabetic nephropathy and membranous nephropathy.

Fifteen percent of the patients required dialysis prior to the biopsy. 58.3% of the patients had complications post-renal biopsy. Five percent of the patients had major complications while 53.33% had minor complications. Thirty percent of the patients had a haematocrit fall of > 10% while 41.25% had a haematoma during the study period. 13.7% patients experienced flank pain, 3.33% had haematuria, 2.08% required a blood transfusion and 1.25% had hypotension requiring IV fluids for resuscitation. A total of 3.33% patients required an additional intervention due to a post-renal biopsy complication. No AV fistulas, nephrectomy, angiographic procedures/surgical procedures or deaths occurred during the course of the study.

Univariate analysis showed that older age, lower eGFR, higher blood urea level, haemoglobin drop at 12 h, haematocrit fall at 6 h and 12 h, reduced renal parenchymal thickness and reduced cortical thickness, biopsy of native kidney, higher grade renal parenchymal changes and more severe IFTA was associated with a complication (Table 1).

**Table 1:** Baseline characteristics

Baseline characteristics (*N* = 240)		Total patients (240)	No complications (*N* = 100)	Complications (*N* = 140)	*P* value
Age (years), mean ± SD		44.62 ± 14.72	42.04 ± 14.75	46.48 ± 14.75	**0.021**
Sex (male), *n* (%)		151 (62.9%)	63 (63%)	88 (62.8%)	0.98
BMI^a^ (kg/m^2^), mean ± SD		24.8 ± 8.145	23.92 ± 4.47	25.43 ± 9.944	0.67
Biopsy done by	Trainee, *n* (%)	204 (85%)	87 (87%)	117 (83.57%)	0.46
	Consultant, *n* (%)	36 (15%)	13 (13%)	23 (16.42%)	
Desmopressin use, *n* (%)		46 (19.2%)	12 (12%)	34 (24.28%)	**0.017**
Biopsy of	Native kidney, *n* (%)	208 (86.7%)	78 (78%)	130 (92.85%)	**0.001**
	Transplant kidney, *n* (%)	32 (13.3%)	22 (22%)	10 (7.14%)	
Systolic blood pressure (mm Hg), mean ± SD		131.93 ± 13.86	130.56 ± 12.74	132.91 ± 14.52	0.19
Diastolic blood pressure (mm Hg), mean ± SD		80 ± 8.76	80.64 ± 8.74	81.86 ± 8.76	0.29
Hypertensives, *n* (%)		186 (77.5%)	72 (72%)	114 (81.42%)	0.08
No. of antihypertensives used, mean ± SD		1.44 ± 1.13	1.11 ± 1.19	1.51 ± 1.08	0.21
Diabetes mellitus, *n* (%)		73 (30.4%)	29 (29%)	44 (31.42%)	0.68
Haemodialysis, *n* (%)		37 (15.4%)	16 (16%)	21 (15%)	0.83
Antiplatelet use, n (%)		24 (10%)	10 (10%)	14 (10%)	1
Serum creatinine (mg/dl), median (IQR)		1.84 (1.04–3.37)	1.57 (0.81–2.3)	2.27 (1.23–4.35)	0.17
Blood urea (mg/dl), median (IQR)		35 (23.25–58)	29.5 (21–43.7)	40 (27–70)	** < 0.001**
24-h urine protein (g), median (IQR)		2.44 (0.995–4.618)	2.39 (1.15–4.22)	2.505 (0.97–4.69)	0.58
Spot UPCR^b^ (g/g), median (IQR)		2.4 (0.931–6.57)	2.21 (0.64–6.57)	2.90 (1–6.9)	0.73
eGFR (ml/min/1.73 m^2^), median (IQR)		42.65 (18.7–74.7)	54.5 (28–98.32)	31.70 (14.15–64)	** < 0.001**
Pre-biopsy haemoglobin (g/dl), mean ± SD		11.46 ± 2.33	11.42 ± 2.27	11.510 ± 2.39	0.79
Haemoglobin at 6 h (g/dl), mean ± SD		10.9 ± 2.29	11.23 ± 2.26	10.70 ± 2.29	0.07
Haemoglobin at 12 h (g/dl), mean ± SD		11.07 ± 2.19	11.47 ± 2.24	10.78 ± 2.12	**0.017**
Pre-biopsy haematocrit %, mean ± SD		34.4 ± 6.7	34.40 ± 6.88	34.40 ± 6.65	0.99
Haematocrit at 6 h %, mean ± SD		32.49 ± 6.34	33.61 ± 6.52	31.70 ± 6.11	**0.022**
Haematocrit at 12 h %, mean ± SD		33.06 ± 6.52	34.38 ± 6.59	32.12 ± 6.33	**0.009**
Pre-biopsy platelets (cells/cc), mean ± SD		267,054.17 ± 90,610	265,120 ± 83,751	268,435.71 ± 95,480.32	0.78
Prothrombin time (pt) (seconds), mean ± SD		10.6 ± 0.81	10.58 ± 0.71	10.71 ± 0.88	0.29
Activated partial thromboplastin time (aptt) (seconds), mean ± SD		28.1 ± 3.4	27.86 ± 3.57	28.41 ± 3.40	0.23
International normalized ratio, mean ± SD		0.9 ± 0.092	0.93 ± 0.067	0.94 ± 0.10	0.65
Bleeding time (min), mean ± SD		3.18 ± 0.65	3.21 ± 0.75	3.19 ± 0.649	0.86
Clotting time (min), mean ± SD		6.38 ± 0.92	6.47 ± 0.99	6.37 ± 0.88	0.53
Mean no. of passes taken, mean ± SD		1.91 ± 0.67	1.83 ± 0.637	1.96 ± 0.704	0.13
Kidney bipolar length (cm), mean ± SD		9.67 ± 0.91	9.78 ± 0.914	9.59 ± 0.90	0.10
Renal cortical thickness (cm), mean ± SD		1.07 ± 0.30	1.26 ± 0.31	1.04 ± 0.288	**0.044**
Renal parenchymal thickness (cm), mean ± SD		1.74 ± 0.40	1.81 ± 0.44	1.70 ± 0.37	**0.037**
Renal parenchymal changes, *n* (%)	Grades 0 and 1	162 (67.5)	76 (76)	86 (61.4)	**0.018**
	Grades 2 and 3	78 (32.5)	24 (24)	54 (38.6)	
No. of glomeruli, mean ± SD		11.02 ± 5.60	11.31 ± 5.08	10.81 ± 5.98	0.49
Adequacy of biopsy, *n* (%)		173 (72.1%)	72 (72%)	101 (72.1%)	0.98
No. of obsolescent glomeruli, median (IQR)		1 (0–4)	1 (0–3)	2 (0–5)	0.09
IFTA^c^, *n* (%)	No/mild	155 (64.5)	74 (74)	81 (57.8)	**0.009**
	Moderate/severe	85 (35.5)	26 (26)	59 (42.2)	

^a^*BMI* body mass index; ^b^*UPCR* urine protein creatinine ratio, *eGFR* estimated glomerular filtration rate, *SD* standard deviation, *IQR* interquartile range; ^c^*IFTA* interstitial fibrosis tubular atrophy

On performing the binary logistic regression of the sonological parameters with the occurrence of complications (Table 2), we found that only renal parenchymal changes were statistically significant (*p* < 0.001) between the groups.

**Table 2:** Univariate and multivariate (logistic regression) analysis of renal sonological parameter

Renal sonological parameters				
Variables	Univariate HR (95% CI)	*P* value	Multivariate HR (95% CI)	*P* value
Renal parenchymal changes	1.988 (1.123–3.521)	0.018*	1.787 (0.995–3.208)	0.034*
Renal cortical thickness	0.412(0.174–0.977)	0.044*	0.643 (0.210–1.970)	0.439
Renal parenchymal thickness	0.503 (0.264–0.959)	0.037*	0.705 (0.306–1.626)	0.412
Bipolar kidney length	0.792 (0.697–1.051)	0.106		

*CI* confidence interval, *HR* hazard ratio; *statistically significant

Ninety-nine patients had haematomas, with 83% of haematomas formed immediately post-renal biopsy while 15% formed by 12 h post-biopsy, i.e. 98.98% of the haematomas were noticed by 12 h post-biopsy (Table 3). Only 1% of the total haematomas was formed between 12–24 h post-renal biopsy. Only 6 haematomas in the study had a size > 5 cm.

**Table 3:** Haematoma profile as per surveillance

Haematoma profile (*n* = 99)	No	Percentage
Total haematoma, *n* (%)	99	100%
Haematoma immediately post-biopsy, *n* (%)	83	83.83%
Total haematoma at 12 h post-biopsy, *n* (%)	88	88.88%
New-onset haematoma at 12 h post-biopsy, *n* (%)	15	15.15%
Resolved haematoma at 12 h post-biopsy, *n* (%)	10	10.10%
Total haematoma at 24 h post-biopsy, *n* (%)	70	70.70%
New-onset haematoma at 24 h post-biopsy, *n* (%)	1	1.01%
Resolved haematoma at 24 h post-biopsy, *n* (%)	19	19.19%
Haematoma > 5 cm in size, *n* (%)	6	6.06%

All the patients with major complications had haematomas. Only 2 patients with major complications had gross haematuria; 6 had flank pain. Five patients required blood transfusion and 3 developed hypotension requiring IV fluids.

On correlating the haematoma size with other biopsy complications—haematoma occurring immediately or by 12 h post-biopsy correlated with major complications needing blood transfusion (*p* = 0.01 and 0 = 0.015 respectively).

**Fig. 3:**
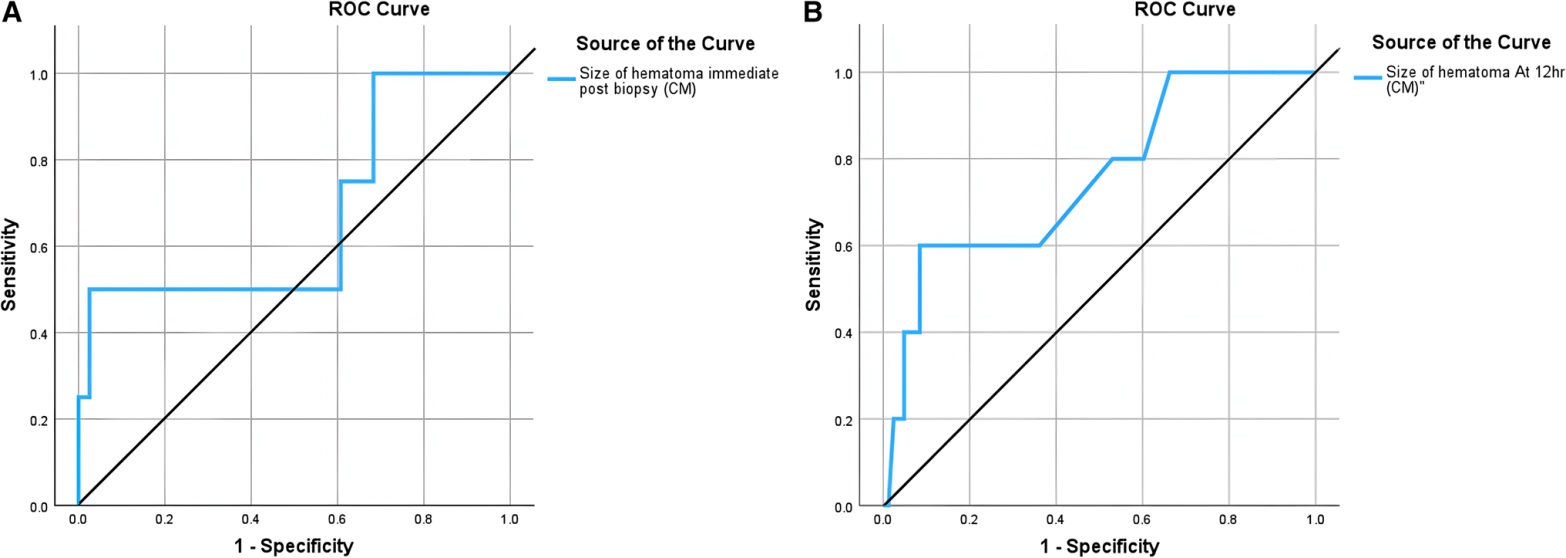
**A** ROC curve for the size of haematoma immediately after renal biopsy for predicting the need for blood transfusion. **B** ROC curve for size of hematoma at 12 h for predicting the need for blood transfusion

The ROC curve for the size of the haematoma immediately post-renal biopsy (Fig. 3A) had AUC of 0.67, with a haematoma size of 1.17 cm showing 100% sensitivity and 74% specificity for predicting the need for blood transfusion.

The ROC curve for the size of the haematoma at 12 h (Fig. 3B) had AUC of 0.75 with a haematoma size of 1.2 cm showing 100% sensitivity and 71% specificity for predicting the need for blood transfusion.

## Discussion

Renal biopsy is an integral part of diagnosis which decides the course of treatment of kidney diseases. However, it is not without risk of complications. Studies on traditional risk factors like elderly age, higher BMI, lower eGFR and presence of coagulation abnormalities have provided insights into making the procedure of kidney biopsy more safe [3–5, 7, 16]. However, very few studies have sought to correlate sonological parameters of the kidney like kidney length, renal cortical and parenchymal thickness, and renal parenchymal changes by ultrasonography—to renal biopsy complication [8, 9]. A limited number of studies have actively surveyed for haematoma and looked for its correlation to biopsy complications. We have, in our present study, examined the correlation of traditional as well as the renal sonological risk factors with post-renal biopsy complications. Furthermore, we conducted an active surveillance for asymptomatic haematomas in order to correlate the size of haematomas with the development of other complications.

Our study has a lower rate of major complications which is comparable to other studies [5, 17–19] including gross haematuria not requiring intervention [16], blood transfusion [4, 17, 20] and hypotension requiring IV fluids for resuscitation [17, 21]. We also observed haematomas in 41.25% of the cases—higher than reported in other studies [16, 17] which resulted in an apparently higher percentage of minor complications in this study. The difference observed could be because other studies only screened for haematomas which were clinically significant, while, in our study, we carried out an active surveillance for haematomas which were, by and large clinically silent. A meta-analysis found that studies which actively screened for haematomas have reported more number of haematomas than studies where active surveillance was not carried out [16]. When CT scans were carried out immediately post-renal biopsy, haematoma was observed in 50% of the cases in such studies [22].

In our study, 83.33% of the haematomas were observed immediately after biopsy, 15.5% new-onset haematomas were observed at 12 h post-biopsy and only 1% of new-onset haematomas were found to occur between 12 and 24 h post-renal biopsy, i.e. a total 99% of the haematomas were detected by 12 h after renal biopsy. This finding is similar to the observations of Simard-Meilleur et al. where 84% of the complications were detected by the first 8 h, 86% by 12 h and 94% by 24 h after renal biopsy [23]. Whittier et al. also found that 85% of the complications are detected at ≤ 12 h post-renal biopsy and 89% by ≤ 24 h [19]. Marwah et al. in their study also found that most of the renal biopsy complications (95%) were detected within 12 h post-renal biopsy [24]. Further correlation of the haematoma size with the risk of development of other renal biopsy complications revealed that size of haematoma of > 1.17 cm immediately post-renal biopsy and > 1.2 cm at 12 h post-renal biopsy were sensitive predictors of a need for blood transfusion; however, the specificity for the same was ~ 70%.

Kidney bipolar length did not show any correlation with increased risk of renal biopsy complication in our study. Many previous studies such as by Roger et al. [25], Jiang et al. [26] and Trajceska et al. [3] reported that a smaller kidney size was associated with higher risk of percutaneous renal biopsy complication. This difference in observation may be due to our strict screening methods, where we deferred from doing biopsies of patients with kidney size < 8.5 cm as they were reflective of kidney with CKD. The mean kidney length in our study was 9.67 ± 0.91 cm. Even among the complications group, the mean kidney length was 9.59 ± 0.90 cm. Renal cortical thickness and renal parenchymal thickness were significantly different among the complications and no complications groups. A study by Zhang et al. found patients with renal parenchymal thickness < 1.5 cm to be at a higher risk for a renal biopsy complication [8]. Mejía-Vilet et al. found that renal cortical thickness < 0.8 cm was associated with chronic renal changes and more likely to have a bleeding complication post-renal biopsy [9]. The study also found that patients with higher grade renal parenchymal changes and poor cortico-medullary differentiations of the kidney on ultrasonography were associated with CKD and were more likely to have a higher risk of renal biopsy complications. In our study, however, on multivariate analysis, there was no significant difference between the no complications and complications groups. This may be due to our stringent screening practices, where we deferred renal biopsy of patients with thinner renal parenchyma or lesser cortical thickness in anticipation of complications. Renal parenchymal changes were statistically different between the complications and no complications groups, with a higher grade of renal parenchymal changes (grade 2 and grade 3) being associated with a higher risk of renal biopsy complication. This could be because a higher grade of renal parenchymal change is associated with chronic renal changes such as seen in CKD and hence, pose greater bleeding risk on biopsy.

Similar to other studies, traditional risk factors like older age, higher blood urea, lower eGFR and native kidney biopsy, haemoglobin fall at 12 h, haematocrit % fall at 6 and 12 h post-biopsy, severe IFTA on biopsy, in our study were indicative of a risk of renal biopsy complication [3, 4, 17, 27–29]. However, gender, BMI, kidney biopsy by trainee/consultant, blood pressure and comorbidities like hypertension/diabetes, pre-biopsy haemoglobin and platelets, proteinuria, coagulation profile, antiplatelet use and number of passes taken were not significantly different between any of the groups.

The strength of our study is that it is one of the few studies to look for association between the renal sonological parameters and the renal biopsy complications. Furthermore, we have carried out an active surveillance for haematomas post-renal biopsy and analysed this for predicting post-renal biopsy complications.

## Conclusion

The rate of major complications post-renal biopsy requiring interventions was low. Renal sonological characteristics like parenchymal thickness, cortical thickness and parenchymal changes may predict post-renal biopsy complications. Haematoma surveillance especially done immediately post-biopsy and at 12 h can be a useful tool to predict the need for intervention post-renal biopsy and triage patients who require further observation.

## Data Availability

The corresponding author of the article can be contacted for need for any data related to the study. It would be made available as per request.
