# Correction to: Sonological predictors of complications of percutaneous renal biopsy—a prospective observational study

**DOI:** 10.1007/s11845-024-03762-x

**Published:** 2024-08-12

**Authors:** Shruti Bhattacharya, Shankar Prasad Nagaraju, Ravindra Attur Prabhu, Dharshan Rangaswamy, Indu Ramachandra Rao, Mohan V. Bhojaraja, Srinivas Vinayak Shenoy

**Affiliations:** Department of Nephrology, Kasturba Medical College, Manipal, Manipal Academy of Higher Education, Manipal, Karnataka 576104 India

**Correction to: Irish Journal of Medical Science (1971 -)**

10.1007/s11845-024-03753-y

The original version of this article unfortunately contained a mistake.

There is an error in the online version of affiliation 1.

Department of Nephrology, Kasturba Medical College, Manipal, Manipal Academy of Higher Education (MAHE), Manipal, India

The correct one is:

Department of Nephrology, Kasturba Medical College, Manipal, Manipal Academy of Higher Education, Manipal, Karnataka 576104, India

The original article has been corrected.

